# Telehealth in children’s psychiatric services

**DOI:** 10.1192/j.eurpsy.2021.929

**Published:** 2021-08-13

**Authors:** O. Shchedrinskaya, M. Bebtschuk, O. Khairetdinov

**Affiliations:** Science, Moscow State Budgetary Health Care Institution “Scientific and Practical Center for Mental Health of Children and Adolescents named after G.E.Sukhareva of Moscow Health Department, Moscow, Russia, Moscow, Russian Federation

**Keywords:** Child Psychiatry, telehealth, quality of services

## Abstract

**Introduction:**

Covid-19 intensified public demand for telehealth services in child psychiatry. The shift towards online services raised concerns related to safety and quality of services.

**Objectives:**

The objective of the study was to explore outcomes and perceptions regarding psychiatric telehealth services from the patients’ and professionals’ perspectives.

**Methods:**

Survey and questionnaires were the main methods to collect feedback after 1129 sessions conducted by psychiatrists and psychotherapists for 559 young patients in 2020.

**Results:**

Overall, patients/caregivers were generally satisfied with the quality of services, despite some technical issues and limitations of the platform. The most common outcomes of the sessions were: psychotherapy, in-depth assessment, pharmacotherapy, in-patient treatment, referrals for in-person appointments with other specialists, parenting strategies. Professionals gave more positive feedback on telehealth services after a few months of practice and training. Psychiatrists preferred conducting telehealth appointments for the patients they have previously seen in-person. The most common diagnosis were various neurodevelopmental disorders (48,9%), as well as patients within F84.0-F84.5 27,9%, and F84.8 (19,8%). Identification challenges, confidentiality and safety maintenance were among the top concerns for mental health workers. Specific guidelines for caregivers helped to use the appointment time effectively, prevent some technical and organizational issues and decrease negative effects of limited communication capabilities during a telehealth appointment.

**Conclusions:**

Telehealth services in psychiatry are meeting real needs of patients, caregivers and professionals, and require further development. Proper training for professionals and clear guidelines for caregivers are among the key factors that enhance the quality of services.

